# Depression, Anxiety, and Neuropsychiatric Symptom Burden in a Longitudinal Cohort with Persistent Psychophysical Post-COVID Olfactory Dysfunction

**DOI:** 10.3390/brainsci14121277

**Published:** 2024-12-19

**Authors:** Tiana M. Saak, Jeremy P. Tervo, Brandon J. Vilarello, Patricia T. Jacobson, Francesco F. Caruana, Matthew D. A. Spence, Liam W. Gallagher, David A. Gudis, Jeffrey N. Motter, Davangere P. Devanand, Jonathan B. Overdevest

**Affiliations:** 1 Vagelos College of Physicans and Surgeons, Columbia University, New York, NY 10032, USA; tms2211@cumc.columbia.edu (T.M.S.);; 2 Department of Otolaryngology-Head and Neck Surgery, University of Miami Miller School of Medicine, Miami, FL 33136, USA; 3 Department of Otolaryngology-Head and Neck Surgery, Yale School of Medicine, New Haven, CT 06510, USA; 4 Department of Otolaryngology-Head and Neck Surgery, University of Pittsburgh, Pittsburgh, PA 15260, USA; 5 Department of Otolaryngology-Head and Neck Surgery, University of Minnesota, Minneapolis, MN 55455, USA; 6 Department of Otolaryngology-Head and Neck Surgery, New York-Presbyterian/Columbia University Irving Medical Center, New York, NY 10032, USA; 7 Department of Psychiatry, New York-Presbyterian/Columbia University Irving Medical Center, New York, NY 10032, USA

**Keywords:** olfactory dysfunction, long COVID, brain fog, depression, anxiety

## Abstract

Background/Objectives: Olfactory dysfunction (OD) is associated with a variety of neurologic deficits and impacts socialization decisions, mood, and overall quality of life. As a common symptom comprising the long COVID condition, persistent COVID-19-associated olfactory dysfunction (C19OD) may further impact the presentations of neuropsychiatric sequelae. Our study aims to characterize the longitudinal burden of depression, anxiety, and neuropsychiatric symptoms in a population with C19OD. Methods: Individuals with perceived C19OD completed a psychophysical screening evaluation of their sense of smell using the comprehensive Sniffin’ Sticks olfactory assessment. Only those with validated psychophysical OD were included in this prospective longitudinal study for baseline and one-year follow-up. Participants also completed PHQ-9, Beck Anxiety Inventory (BAI), and neuropsychiatric symptom questionnaires at each time point. Anxiety, depression, and neuropsychiatric symptom prevalence was calculated and compared between time points with Pearson’s chi-squared, Fisher’s exact, and Wilcoxon rank sum tests. Results: Each neuropsychiatric symptom evaluated in this study was reported by 13–49% of longitudinal cohort participants at both baseline and follow-up, except for seizure (0% at baseline and follow-up) and word-finding difficulty (61–68% at baseline and follow-up). Word-finding and focus difficulties were the most commonly reported symptoms. In total, 41% of participants reported some level of depression at baseline and 38% of participants reported depression at one-year follow-up, while 29% and 27% of participants reported some level of anxiety at respective time points. Conclusions: Individuals with C19OD are at risk for developing persistent neuropsychiatric conditions. These neurologic and psychiatric sequelae are persistent with repeated longitudinal assessment, even at nearly 2.5 years following initial COVID-19 diagnosis.

## 1. Introduction

There is a long-standing relationship between olfactory dysfunction (OD) and neurodegenerative diseases including Alzheimer’s disease [1,2,3] and Parkinson’s disease [3], in addition to the prevalence of OD in neuropsychiatric and mood disorders [4,5] such as schizophrenia [6]. Alteration in olfaction is one of the earliest signs of neurodegeneration, often occurring years before structural disease progression [1,7,8]. Further, structural and functional studies [9,10,11] suggest OD may exacerbate and contribute to depression [12], anxiety [13,14], and olfactory perceptions associated with certain epileptic conditions [15].

The relationship between OD and neuropsychiatric conditions is one of interest among both public and research communities following the COVID-19 pandemic. Early in the pandemic, smell loss was one of the key indicators of SARS-CoV-2 infection [16,17], and it has colloquially established itself to be a defining feature. Estimates suggest that 6.9% of adults have “long COVID”, a condition defined by persistent COVID symptoms more than three months following diagnosis [18]. Both quantitative (hyposmia, anosmia, hypogeusia, and ageusia) and qualitative (phantosmia, parosmia, phantogeusia, and parogeusia) smell and taste dysfunctions are classic symptoms of long COVID, with nearly a third of patients who recover from COVID-19 reporting persistent qualitative smell/taste dysfunction [19] and approximately a quarter to a third of patients reporting measurable quantitative OD for months after infection [17]. Symptoms of long COVID extend beyond olfaction to multiple organ systems [20] and include a variety of neurologic and psychiatric consequences such as cognitive impairment or brain fog and symptoms of anxiety and depression [21,22,23,24,25]. A large retrospective study of nearly 1.3 million patients tracking neurologic and psychiatric risk following SARS-CoV-2 infection—without accounting for olfaction status—found that the COVID-related increased incidence of anxiety and depression tapered off after an initial spike, though there was a persistent increased risk of other neuropsychiatric disorders including psychosis, cognitive deficit, and dementia [26].

The mental health burden of long COVID and OD is complicated and extensive [25,27]. COVID-19-associated olfactory dysfunction (C19OD) potentially contributes to the varied neuropsychiatric presentations in long COVID and OD, thus requiring a systematic biopsychosocial investigative approach [17,28]. Pre-existing biological relationships between olfaction and neuropsychiatric disorders paired with the distinct social and psychological stressors introduced by the pandemic and abrupt loss of smell [29,30] pose a unique challenge in teasing out the associations between C19OD and neuropsychiatric disease in a young cohort. While studies examined neuropsychiatric conditions following self-reported smell alterations [31,32,33,34], with one study examining anxiety and depression symptoms among individuals with acute smell loss [35], neuropsychiatric outcomes among those with persistent C19OD remain limited.

Thus, the objective of this prospective cohort study is to evaluate the prevalence and changes in anxiety, depression, and neuropsychiatric symptoms longitudinally in a long COVID population comprised entirely of individuals with quantitative psychophysical testing-confirmed C19OD.

## 2. Materials and Methods

A total of 145 interested individuals were screened for enrollment. After applying inclusion criteria, 97 participants were enrolled in the study for baseline neuropsychiatric symptom reporting and olfactory psychophysical assessment, with 48 participants following up at one year. Individuals were recruited and evaluated in accordance with the proposed protocols (AAAU5380) approved by the Columbia University Irving Medical Center Institutional Review Board. Participants were recruited via referral from clinicians at Columbia University Irving Medical Center for COVID-related smell loss and the Columbia University online research recruitment platform. Informed consent was retrieved from interested individuals prior to participation in the study.

To participate in the study, the following inclusion criteria were applied: age ≥ 18 years, confirmed SARS-CoV-2 positivity via PCR or SARS-CoV-2 nucleocapsid antibody serology, self-reported persistent OD (>3 months), and OD confirmed by psychophysical testing. Clinical diagnosis of COVID-19 was accepted if the participant was diagnosed prior to widespread testing availability. OD was defined as a Sniffin’ Sticks TDI score ≤ 30.5, indicating the presence of anosmia or hyposmia (classified as quantitative dysfunction) [36]. Exclusion criteria included any diagnosed pre-existing olfactory dysfunction, SNOT-22 rhinologic subdomain score ≥ 21 as assessed on pre-evaluation surveys, any pre-existing neurologic or other health problems than can independently cause olfactory dysfunction, and any self-reported pre-existing functional cognitive deficit.

Prior to in-person evaluation, participants completed surveys to report any neuropsychiatric or neurologic symptom appearance or worsening since SARS-CoV-2 infection. For each symptom assessed, participants were given the option to select “Yes”, “No”, or “Unsure”. Further, participants completed the validated Beck Anxiety Inventory (BAI) [37,38] and Patient Health Questionnaire-9 (PHQ-9) [39] screening instruments to detect and measure symptoms associated with anxiety and depression. They also completed surveys assessing patient demographics, COVID-19 history, and perception of COVID illness severity.

Olfactory assessment was completed by a trained research assistant using the validated extended Odofin Sniffin’ Sticks test (Burghart Messtechnik GmBH, Holm, Germany), including odor threshold (T), odor discrimination (D), and odor identification (I). Measurement of T, D, and I and the combined TDI score was completed following the initial protocol established by Hummel et al. (1997) [40] and repeated in other studies examining various neurologic outcomes [41,42]. The Sniffin’ Sticks test uses felt-tipped pens containing odorants dissolved in propylene glycol. Each felt-tipped pen was placed 2 cm in front of the participant’s nostrils for 3 s. For threshold testing, the subject was asked to identify the odorant-containing pen among a triplet with a gradually increasing concentration of the odorant. For discrimination, the subject selected the unique odorant out of a triplet containing two identical odorants over 16 trials of triplets. Finally, for the identification test, subjects were presented with 16 common odors which they were asked to identify out of 4 multiple choice options. Individual scores for T, D, and I range from 1 to 16, and the sum of the individual scores comprise the TDI score. Individuals are deemed normosmic, hyposmic, or anosmic based on cutoffs of >30.5, 16.5–30.5, and <16.5, respectively [43].

IBM SPSS Statistics for macOS, version 29.0 (IBM Corp., Armonk, NY, USA) was used for all statistical analyses. Neuropsychiatric and neurologic symptom prevalence was calculated both at baseline and at one-year follow-up. To account for any potential bias in longitudinal analysis due to loss to follow-up, participants were stratified by follow-up status, and a comparison of baseline characteristics including age, sex, education status, BAI, PHQ-9, and TDI was completed using the Wilcoxon rank sum test. Among participants completing both assessments (longitudinal cohort), a paired longitudinal analysis of prevalence for each symptom between time points was assessed using the McNemar test. Further, the change in symptom status for each neuropsychiatric and neurologic symptom was reported.

We used BAI and PHQ-9 screening instruments to identify individuals with symptoms associated with anxiety [37] and depression [39]. Individuals were stratified by severity using cutoffs for BAI score (no anxiety: 0–7; mild: 8–15; moderate: 16–25; and severe: 26–63) and PHQ-9 score (no depression: 0–4; mild: 5–9; moderate: 10–14; moderately severe: 15–19; and severe: 20–27), respectively, for both the full study population at baseline and for the longitudinal cohort at each time point. Among the longitudinal cohort, a paired analysis was completed to compare (1) median BAI and PHQ-9 scores and (2) stratification by anxiety and depression severity between time points using the Wilcoxon signed rank test. Comparisons of neuropsychiatric symptom prevalence, BAI, and PHQ-9 scores between genders were completed via Pearson’s chi-squared, Fisher’s exact, and Wilcoxon rank sum tests at each time point.

TDI scores for participants were reported for the full study population at baseline and the longitudinal cohort at each time point. A paired longitudinal analysis of TDI score evolution was completed using the Wilcoxon signed rank test, with paired analyses allowing for comparison of each TDI subdomain between time points as well. Further, TDI scores were correlated against BAI and PHQ-9 scores at baseline and follow-up using Spearman’s bivariate correlations.

## 3. Results

### 3.1. Cohort Demographics

Baseline surveys, neuropsychiatric/neurologic questionnaires, and psychophysical olfactory assessments were performed for 97 participants (full study population), of which 71 (73%) were female and 26 (27%) were male (Table 1). Of these, 48 participants returned to follow-up at one year (longitudinal cohort) to complete olfactory assessment and repeated neuropsychiatric/neurologic questionnaires, including 32 (67%) females and 16 (33%) males. Baseline testing was performed at a median 466 days from COVID-19 diagnosis, while one-year follow-up testing was performed at a median 888 days from COVID-19 diagnosis. Most participants self-reported the perceived severity of their COVID-19 illness as substantially (15%) or slightly (35%) less severe than others at both time points. The median age of participants was 42 years old at baseline testing and 41 years old at one-year follow-up, where 26 (27%) of the cohort self-identified as Hispanic or Latino/a.

At baseline assessment, the lost group to follow up was not found to significantly differ from the longitudinal cohort in age (*p* = 0.4), sex (*p* = 0.058), education status (*p* = 0.7), PHQ-9 score (*p* = 0.5), or BAI score (*p* = 0.3) (Table S1) upon performing Pearson’s chi-squared (sex) and Wilcoxon rank sum tests (age, education status, PHQ-9, and BAI). However, at baseline assessment, the longitudinal cohort had a significantly higher TDI score (median [IQR]: 26 [20.63, 28.63]) than the group that was lost to follow up (22.5 [19, 27]) (*p* = 0.034), though both groups’ medians and IQRs were within the hyposmic range. For the full study population, median (IQR) threshold score was 5 (2, 7.5), median (IQR) discrimination was 10 (8, 11), and median (IQR) identification score was 9 (7, 10) (Table S2), while the median (IQR) threshold, discrimination, and identification scores were 5.75 (2.25, 7.56), 10 (8, 11.25), and 9 (8, 11), respectively, for the longitudinal cohort at baseline assessment (Table S3).

### 3.2. Neuropsychiatric and Neurologic Symptom Prevalence at Baseline and Follow-Up

Prevalence of neuropsychiatric symptoms and neurologic conditions reported by longitudinal cohort participants, along with the rates of amelioration or worsening over time are reported in Table 2. Prevalence of symptoms for full study population at baseline is reported in Table S2. Among the longitudinal cohort at baseline testing, the range of each neuropsychiatric symptom prevalence was 23–49% of participants, with the rate of word-finding difficulty and seizures outside of this at 61% and 0%, respectively. At least one symptom was reported by 38 (79%) participants. The most prevalent symptoms were word-finding difficulty (61%) and difficulty staying focused (49%), followed by difficulty thinking clearly (39%); difficulty remembering conversations (35%); difficulty remembering object placement (34%); headache (30%); vision changes (26%); dizziness, imbalance, or vertigo (24%); numbness (24%); weakness; and seizure (0%).

At follow-up, the range of each symptom prevalence was 13–46% of participants, other than word-finding difficulty and seizures. At least one symptom was reported in 33 (76%) of all individuals following up after one year. The most prevalent symptoms at follow-up were word-finding difficulty (68%) and difficulty staying focused (46%), followed by the rest of the symptoms: difficulty thinking clearly (39%); difficulty remembering conversations (35%); headache (30%); difficulty remembering object placement (29%); dizziness or vertigo (29%); numbness (24%); weakness (21%); vision changes (13%); and seizures (0%).

### 3.3. Change in Neuropsychiatric and Neurologic Symptoms over Time

For all participants who followed up at one year (n = 48), the change in status for each symptom was tallied, as presented in Table 2. For most participants, the tabulated change in symptom status was “no change”, meaning most participants reported the presence or absence of each symptom at one-year follow-up as they did at baseline testing. Further, there were no neuropsychiatric or neurologic symptoms that were found to be significantly more prevalent at either time point (Table 2).

### 3.4. Anxiety and Depression Among Participants

Among the longitudinal cohort at baseline, 12 (29%) of participants who responded to the BAI assessment were found to have some level of clinical anxiety: mild (12%); moderate (12%); and severe (5%); at one-year follow-up, 11 (27%) of responding participants were found to have some level of clinical anxiety: mild (20%); moderate (5%); and severe (2%) (*p* = 0.5) (Table 3). The median (IQR) BAI scores at baseline and one-year follow-up were 4 (1, 10.5) and 4 (1, 10), respectively (*p* = 0.4).

At baseline testing, 19 (41%) participants in the longitudinal cohort who responded to the PHQ-9 assessment were found to have some level of clinical depression: mild (19%); moderate (11%); moderately severe (9%); and severe (2%); at one-year follow-up, 15 (38%) were found to have some level of clinical depression: mild (20%), moderate (5%), moderately severe (5%), and severe (5%) (*p* > 0.9) (Table 3). The median (IQR) PHQ-9 scores at baseline and follow-up were 3 (1, 8) and 3 (1, 7.5), respectively (*p* = 0.8).

Among the full study population, 27 (33%) participants were found to have some level of clinical anxiety at baseline, while 37 (37%) participants were found to have some level of clinical depression (Table S2). Further BAI and PHQ-9 median scores and stratification by severity for the full study population at baseline were reported in Table S2. 

At baseline and follow-up assessment, TDI score was not found to correlate with BAI (ρ [CI] significance at respective time points: −0.041 [−0.353, 0.259], 0.8, and −0.185 [−0.488, 0.135], 0.3) or PHQ-9 (0.057 [−0.300, 0.390], 0.7, and −0.222 [−0.529, 0.133], 0.2), though TDI score for all individuals included in the study had a score ≤30.5, indicating the presence of hyposmia or anosmia (Table S4).

### 3.5. Neuropsychiatric Symptom Status, Anxiety, and Depression by Gender

Among the full study population, women (N = 71) reported significantly higher rates than men (N = 26) of many neuropsychiatric symptoms including difficulty remembering placement of objects (% men, % women, significance: 10%, 36%, and *p* = 0.021), headaches (10%, 34%, and *p* = 0.022), and dizziness or vertigo (13%, 35%, and *p* = 0.039) (Table S5). At follow-up, women (N = 32) reported a significantly higher rate of word-finding difficulty than men (N = 16) (50%, 80%, and *p* = 0.048). There were no significant differences between men and women on any of the other symptoms at baseline or follow-up. Further, there were no significant differences in BAI or PHQ-9 score by gender at baseline or follow-up.

### 3.6. Quantitative, Psychophysical Olfactory Status at Baseline and Follow-Up

At baseline, all participants had psychophysical OD as determined by Sniffin’ Sticks TDI score in accordance with the inclusion criteria of the study. At one-year follow-up (n = 48), 30 (68%) participants had persistent OD, while 18 (32%) had recovered to a level of normosmic olfactory function, represented by a TDI score > 30.5. Among the longitudinal cohort, there was significant TDI score improvement over time from a median (IQR) score of 26 (20.63, 28.63) to 28.75 (24, 33) (*p* < 0.001) (Table S3). In olfactory subdomains, there was significant improvement over time on a threshold from 5.75 (2.25, 7.56) to 7.75 (5.25, 10) (*p* = 0.002) and discrimination from 10 (8, 11.25) to 11 (10, 12) (*p* = 0.028). However, there was no significant difference seen in identification score between the time points (*p* = 0.208).

## 4. Discussion

This longitudinal cohort study aimed to characterize the burden of neuropsychiatric symptoms, depression, and anxiety among individuals with persistent psychophysical C19OD. While patients with olfactory deficits have an increased risk of neurologic and psychiatric disease and dysfunction [6,44,45,46], the longitudinal implications of persistent OD following sudden virally mediated smell loss remain less understood. Further, the COVID-19 pandemic introduced a large mental health burden, with many neurologic and psychiatric symptoms reported in long COVID populations [17,22,23,24,25,47,48]. We sought to examine these overlapping neuropsychiatric, olfactory, and COVID-19 relationships longitudinally in this unique, young population with persistent OD and long COVID following SARS-CoV-2 infection.

The neuropsychiatric symptom burden in the population reported here is substantial, with approximately 70% of all participants reporting at least one symptom at baseline and 76% at one-year follow-up. Word-finding difficulty and difficulty staying focused were the most prevalent symptoms at both baseline and one-year follow-up, with nearly 2/3 of longitudinal cohort participants reporting word-finding difficulty at each time point, and nearly half reporting difficulty staying focused. Most other symptoms (except seizure and vision change) were reported in approximately 20–35% of participants at both baseline and follow-up. Much of the neuropsychiatric symptom prevalence reported in this study can be attributed to women reporting significantly higher rates than men of difficulty remembering placement of objects, headaches, and dizziness or vertigo at baseline and a higher prevalence of word-finding difficulty at follow-up (Table S4). Female gender has been independently associated with long COVID [49], which may explain both the overrepresentation of women included in the study and their higher reported prevalence of neuropsychiatric and neurologic symptoms.

Importantly, most participants failed to experience a change in their symptom status between baseline and follow-up. That is, those who were not experiencing the symptom initially were unlikely to develop the symptom later, while those who were experiencing the symptom at baseline were unlikely to experience recovery. In this study, the median time for follow-up evaluation was 888 days after an initial COVID diagnosis, representing a striking persistence for these neuropsychiatric and neurologic symptoms nearly 2.5 years following illness.

Many of these symptoms can be characterized as features of “brain fog”, a term often used by patients to describe a perception of cognitive impairment. This description typically conveys a variety of symptoms, including concentration difficulty, confusion, cognitive slowing, forgetfulness, word-finding difficulty, short-term memory loss, and mental fatigue, and is especially relevant in the context of long COVID [50,51,52]. The World Health Organization (WHO) defines brain fog as a ‘clouding of consciousness’ and recognizes its significance in long COVID [53]. A large meta-analysis found the prevalence of brain fog to be 23% among long COVID patients, using reports of concentration difficulties, irritability, memory loss, and/or confusion to describe the phenomenon [52]. The two most prevalent symptoms described in our C19OD population, word-finding difficulty and difficulty staying focused, are classic presentations of brain fog and found in approximately 1/2 to 2/3 of our study population.

Word-finding difficulty is a symptom that may reflect primary language deficits or may emerge secondarily to deficits in other cognitive domains, and occurs in a variety of neuropsychiatric disorders, including dementias and mood disorders [54]. It is often associated with the left posterior temporal regions of the brain [55,56]. Meanwhile, difficulty staying focused or concentration difficulty can be described as part of the attention domain of cognition with executive functioning components [57]. Other cognitive domains implicated by the prevalent symptoms reported by patients here include memory (remembering placement of objects and conversations) and executive function (difficulty thinking clearly). Many of these domains, particularly memory, executive function, and concentration/attention are associated with the prefrontal cortex [58], an area also heavily implicated in olfaction [4], providing a potential structural basis for the reported functional deficits in this C19OD population [42].

The prevalence of anxiety and depression in long COVID patients is equivalent for both disorders at 23%, according to a large meta-analysis of more than 7.7 million and 9.3 million patients, respectively [47]. In the long COVID population reported herein, exclusively of individuals with psychophysical OD, 29% of participants at baseline, and 27% at follow-up were found to have some level of anxiety, while 40% of participants at baseline and 38% at follow-up were found to have some level of depression (Table 3). It can be difficult to compare these values considering the wide varieties of neuropsychiatric screening and assessment tools utilized across the meta-analysis and this study. However, among the long COVID population, notably those with C19OD as included in our study, many experience high rates of depressive and anxious symptoms that appear to persist well beyond the onset of the original illness.

Importantly, the prevalence of anxiety and depression in the general adult population just before the COVID-19 pandemic was 8.2% and 6.6%, respectively, as assessed by positive screens on the PHQ-2 and GAD-7, according to the 2019 U.S. Census Bureau [59]. It can be difficult to differentiate the origin of the increased burden of anxiety and depression, given the competing biological, psychological, and social forces of the mental health crisis during and following the pandemic. It is perhaps most useful to adopt a biopsychosocial approach in interpreting these results. Unique social stresses of the pandemic, combined with psychological distress and isolation, contributed to the increased prevalence of anxiety and depression. While the initial spike in anxiety and depression found in the general population at the beginning of the pandemic eventually tapered [26], this resolution does not appear to occur among those with C19OD and long COVID.

Despite a significant improvement in TDI score over time, particularly within the threshold and discrimination domains (Table S5), the majority of participants were still experiencing psychophysical OD at follow-up assessment. The lack of significant correlation between TDI and PHQ-9 or BAI score (Table S3) suggests that severity of OD likely does not meaningfully modify the existing relationship between OD and neuropsychiatric disease in the C19OD population. This is further supported by the persistence of PHQ-9 and BAI score over time despite some improvement in TDI score (Table 3). Therefore, it is likely that the presence of COVID-related OD alone, regardless of severity, has a meaningful impact on neuropsychiatric symptom development and mental health.

C19OD continues to pose a large public health concern. Moving forward, it is important to engage health professionals across primary care, otolaryngology, neurology, and psychiatry to mitigate the risks and severity of these symptoms while working towards interdisciplinary interventions and solutions. Prior research from our group found that among patients with C19OD, approach and engagement coping mechanisms have the potential to improve depression, while avoidant and disengagement coping mechanisms are associated with worse mental health and quality of life outcomes in this population [60]. A variety of integrated approaches have been used to address different elements of patient health and quality of life, including pharmacologic treatment and dietary behavior counseling in an attempt to curtail long COVID neuropsychiatric challenges and patient mental health [61]. Further, olfactory enrichment, commonly referred to as olfactory training, may contribute to the improvement of C19OD and thus ameliorate associated depressive symptoms [62]. Taken together, these offer several mechanisms for lessening the burden of mental health disturbances in C19OD.

This study has several strengths, including (1) validated measures for assessing anxious and depressive symptoms; (2) validated psychophysical olfactory assessment using the extended Sniffin’ Sticks battery to encompass several smell modalities in one TDI score; (3) using TDI scores for determination of olfactory dysfunction status as many other studies rely on self-report; (4) longitudinal assessment of the relationship between neuropsychiatric symptoms and disorders and psychophysical olfactory dysfunction; and (5) young mean age of 42, minimizing the risk for confounding by age-related neuropsychiatric and/or olfactory decline.

Our study also has limitations. We rely on the BAI and PHQ-9 instruments, which are validated screening tools for anxiety and depression, but lack the capability of conferring a confirmatory neuropsychiatric clinical diagnosis. While our overall study group is comprised of 97 individuals who were eligible for inclusion following a time-consuming psychophysical olfactory testing battery, subgroup analyses were limited by small participant numbers, further exacerbated by the lack of availability of all participants at the one-year follow-up. To mitigate potential biases that loss to follow-up may have introduced, we completed an analysis comparing baseline results of various demographic factors and outcomes relevant to our study with participants stratified by follow-up status (Table S1). We found that the groups had no significant differences in age, sex, education status, PHQ-9, or BAI score at baseline, though the longitudinal cohort did have higher baseline TDI scores than the group that was lost to follow-up. Both groups had TDI score medians within the hyposmic range, consistent with typical OD presentation in C19OD [17]. Since those who were lost to follow up were similar to those who returned for both assessments, we concluded that the results obtained by paired analyses reported throughout this manuscript for the longitudinal cohort are likely representative of our full study population. We also report the baseline results for all outcomes of interest for the full study population (N = 97) in Table S2, as the overall prevalence for neuropsychiatric symptoms, anxiety, and depression, is a primary aim of this study. While this study was a prospective longitudinal study primarily aimed at quantifying the prevalence of neuropsychiatric symptom and disease burden within a population, it would have been ideal to have a normative non-COVID control group to follow longitudinally. This was not possible given the study design, available resources, and difficulty in confirming lack of SARS-CoV-2 exposure. While not a perfect comparison given varying methodologies and study designs, our discussion of the reported prevalence of anxiety and depression in the long COVID population (with or without OD) [47] and normative pre-COVID adult prevalence of anxiety and depression [59] provides important context to the findings reported in this study. Lastly, while comprehensive Sniffin’ Sticks TDI evaluation is a common and validated way to assess a patient’s sense of smell against a normative sample, it does not always reflect an individual’s lived experience [63].

## 5. Conclusions

Patients with psychophysically verified C19OD are seemingly at elevated risk for developing neuropsychiatric symptoms, anxiety, and depression. Word-finding difficulty and difficulty staying focused are among the most commonly reported symptoms in this population, with women reporting many symptoms at higher rates than men. These neurologic and psychiatric sequelae remain persistent with repeated longitudinal assessment, even at nearly 2.5 years following initial COVID-19 diagnosis, suggesting the pervasive and ongoing mental health burden in the C19OD population. Further, these symptoms are persistent regardless of relative improvement in the severity of OD. There is a need for further rigorous longitudinal assessment in the long COVID population to continue characterizing neuropsychiatric post-acute sequelae of COVID-19 and persistent olfactory dysfunction extending beyond 2–3 years following diagnosis.

## Figures and Tables

**Table 1:** Demographics and descriptive statistics.

	Full Study Population, N = 97 ^1^	Longitudinal Cohort, N = 48 ^1^
Age, median (IQR)	42 (33, 58)	41 (33, 56)
**Time from COVID-19 diagnosis**		
Baseline, median days (IQR)	466 (22, 643)	490 (299, 613)
Follow-up, median days (IQR)	--	888 (732, 1010)
**Gender**		
Women	71 (73%)	32 (67%)
Men	26 (27%)	16 (33%)
**Ever Smoked**	18 (19%)	9 (19%)
Not reported or unsure	1	0
**Active smoking**		
Daily	0 (0%)	0 (0%)
Less than daily	2 (2%)	1 (2%)
None	93 (96%)	46 (96%)
Not reported	2 (2%)	1 (2%)
**Ethnicity**		
Hispanic or Latino/a	26 (27%)	12 (25%)
Not Hispanic or Latino/a	67 (69%)	35 (73%)
Prefer not to respond	4 (4%)	1 (2%)
**Severity of COVID-19 illness**		
Slightly less severe	34 (35%)	16 (33%)
Substantially less severe	15 (15%)	9 (19%)
About the same as others	15 (15%)	11 (23%)
Slightly more severe	18 (19%)	7 (15%)
Substantially more severe	6 (6%)	2 (4%)
Not reported	9 (9%)	3 (6%)

^1^ n (%).

**Table 2:** Neuropsychiatric and neurologic symptom prevalence and evolution over longitudinal assessment among participants who completed assessment at both time points (N = 48).

Neuropsychiatric Symptom	Baseline ^1^*	One-Year ^1^*	*p*-Value ^2^	No Change at Follow-Up ^1^	Symptom Improved ^1^	Symptom Worsened ^1^
**Since your COVID illness, do you now have more difficulty…**						
**Remembering conversations a few days later?**				26 (65%)	7 (18%)	7 (18%)
Yes	14 (35%)	14 (35%)	>0.9			
No	26 (65%)	26 (65%)				
**Remembering placement of familiar objects?**				28 (74%)	4 (11%)	6 (16%)
Yes	13 (34%)	11 (29%)	0.75			
No	25 (66%)	27 (71%)				
**Finding the right words when speaking?**				29 (76%)	6 (16%)	3 (8%)
Yes	23 (61%)	26 (68%)	0.51			
No	15 (39%)	12 (32%)				
**Thinking clearly?**				32 (84%)	3 (8%)	3 (8%)
Yes	15 (39%)	15 (39%)	>0.9			
No	23 (61%)	23 (61%)				
**Staying Focused?**				28 (76%)	4 (11%)	5 (14%)
Yes	18 (49%)	17 (46%)	>0.9			
No	19 (51%)	20 (54%)				
**Since your COVID illness, have you** **experienced…**						
**New or more frequent/severe headaches?**				28 (70%)	6 (15%)	6 (15%)
Yes	12 (30%)	12 (30%)	>0.9			
No	28 (70%)	28 (70%)				
**Weakness in one or more parts of your body?**				32 (82%)	3 (8%)	4 (10%)
Yes	9 (23%)	8 (21%)	>0.9			
No	30 (77%)	31 (79%)				
**Numbness or tingling?**				35 (85%)	3 (7%)	3 (7%)
Yes	10 (24%)	10 (24%)	>0.9			
No	31 (76%)	31 (76%)				
**Changes in vision?**				32 (82%)	1 (3%)	6 (15%)
Yes	10 (26%)	5 (13%)	0.13			
No	29 (74%)	34 (87%)				
**Sense of dizziness, imbalance, or vertigo?**				37 (90%)	3 (7%)	1 (2%)
Yes	10 (24%)	12 (29%)	0.63			
No	31 (76%)	29 (71%)				
**Seizures?**				41 (100%)	0	0
Yes	0 (0%)	0 (0%)	>0.9			
No	41 (100%)	41 (100%)				

^1^ n (%). ^2^ McNemar Test. * total participants reported for each symptom will not equate to 48 due to some participants choosing: (1) to not reply; or (2) “unsure”.

**Table 3:** Prevalence and longitudinal evolution of anxiety and depression stratified by severity and median BAI and PHQ-9 scores among participants who completed both baseline and follow-up assessment.

	Baseline ^1^	One-Year ^1^	*p*-Value ^2^
**Beck Anxiety Inventory (BAI)**			0.48
No anxiety	31 (72%)	30 (73%)	
Mild anxiety	5 (12%)	8 (20%)	
Moderate anxiety	5 (12%)	2 (5%)	
Severe anxiety	2 (5%)	1 (2%)	
No response	15	7	
**PHQ-9**			0.97
No depression	28 (60%)	25 (63%)	
Mild depression	9 (19%)	8 (20%)	
Moderate depression	5 (11%)	3 (8%)	
Moderately severe depression	4 (9%)	2 (5%)	
Severe depression	1 (2%)	2 (5%)	
No response	6	8	
**Overall Median (IQR) Scores**			
BAI	4 (1, 10.5)	4 (1, 10)	0.37
PHQ-9	3 (1, 8)	3 (1, 7.5)	0.75

^1^ n (%); median (IQR). ^2^ Wilcoxon signed rank test.

## Data Availability

The original contributions presented in this study are included in the article. Further inquires can be directed to the corresponding author.

## Supplementary Materials

The following supporting information can be downloaded at: https://www.mdpi.com/article/10.3390/brainsci14121277/s1, Table S1: Characteristics and baseline scores of those who completed assessments at both time points (longitudinal cohort) versus those who were lost to follow up; Table S2: Prevalence of neuropsychiatric symptoms, depression, anxiety, and median BAI, PHQ-9, and TDI scores at baseline among all study participants (including those lost to follow up); Table S3: TDI median scores and evolution among longitudinal cohort (N = 48); Table S4: Correlations of TDI score versus BAI and PHQ-9 scores at baseline and follow-up among longitudinal cohort; Table S5: Prevalence of neuropsychiatric symptoms, anxiety, and depression by gender at each time point.

## References

[1] Kotecha A.M., Correa A.D.C., Fisher K.M., Rushworth J.V. (2018). Olfactory Dysfunction as a Global Biomarker for Sniffing out Alzheimer’s Disease: A Meta-Analysis. Biosensors.

[2] Silva M.D.M.E., Mercer P.B.S., Witt M.C.Z., Pessoa R.R. (2018). Olfactory Dysfunction in Alzheimer’s Disease: Systematic Review and Meta-Analysis. Dement. Neuropsychol..

[3] Marin C., Vilas D., Langdon C., Alobid I., Lopez-Chacon M., Haehner A., Hummel T., Mullol J. (2018). Olfactory Dysfunction in Neurodegenerative Diseases. Curr. Allergy Asthma Rep..

[4] Yang K., Ayala-Grosso C., Bhattarai J.P., Sheriff A., Takahashi T., Cristino A.S., Zelano C., Ma M. (2023). Unraveling the Link between Olfactory Deficits and Neuropsychiatric Disorders. J. Neurosci..

[5] Atanasova B., Graux J., El Hage W., Hommet C., Camus V., Belzung C. (2008). Olfaction: A Potential Cognitive Marker of Psychiatric Disorders. Neurosci. Biobehav. Rev..

[6] Moberg P.J., Kamath V., Marchetto D.M., Calkins M.E., Doty R.L., Hahn C.-G., Borgmann-Winter K.E., Kohler C.G., Gur R.E., Turetsky B.I. (2014). Meta-Analysis of Olfactory Function in Schizophrenia, First-Degree Family Members, and Youths At-Risk for Psychosis. Schizophr. Bull..

[7] Adams D.R., Kern D.W., Wroblewski K.E., McClintock M.K., Dale W., Pinto J.M. (2018). Olfactory Dysfunction Predicts Subsequent Dementia in Older, U.S. Adults. J. Am. Geriatr. Soc..

[8] Fullard M.E., Morley J.F., Duda J.E. (2017). Olfactory Dysfunction as an Early Biomarker in Parkinson’s Disease. Neurosci. Bull..

[9] Takahashi T., Nishikawa Y., Yücel M., Whittle S., Lorenzetti V., Walterfang M., Sasabayashi D., Suzuki M., Pantelis C., Allen N.B. (2016). Olfactory Sulcus Morphology in Patients with Current and Past Major Depression. Psychiatry Res. Neuroimaging.

[10] Kiparizoska S., Ikuta T. (2017). Disrupted Olfactory Integration in Schizophrenia: Functional Connectivity Study. Int. J. Neuropsychopharmacol..

[11] Zhou G., Lane G., Cooper S.L., Kahnt T., Zelano C. (2019). Characterizing Functional Pathways of the Human Olfactory System. eLife.

[12] Kohli P., Soler Z.M., Nguygen S.A., Muus J.S., Schlosser R.J. (2016). The Association Between Olfaction and Depression: A Systematic Review. Chem. Senses.

[13] Chen X., Guo W., Yu L., Luo D., Xie L., Xu J. (2021). Association between Anxious Symptom Severity and Olfactory Impairment in Young Adults with Generalized Anxiety Disorder: A Case–Control Study. Neuropsychiatr. Dis. Treat..

[14] Takahashi T., Itoh H., Nishikawa Y., Higuchi Y., Nakamura M., Sasabayashi D., Nishiyama S., Mizukami Y., Masaoka Y., Suzuki M. (2015). Possible Relation between Olfaction and Anxiety in Healthy Subjects. Psychiatry Clin. Neurosci..

[15] Desai M., Agadi J.B., Karthik N., Praveenkumar S., Netto A.B. (2015). Olfactory Abnormalities in Temporal Lobe Epilepsy. J. Clin. Neurosci..

[16] Yan C.H., Faraji F., Prajapati D.P., Boone C.E., DeConde A.S. (2020). Association of Chemosensory Dysfunction and COVID-19 in Patients Presenting with Influenza-like Symptoms. Int. Forum Allergy Rhinol..

[17] Doty R.L. (2022). Olfactory Dysfunction in COVID-19: Pathology and Long-Term Implications for Brain Health. Trends Mol. Med..

[18] Adjaye-Gbewonyo D., Vahratian A., Perrine C.G., Bertolli J. (2023). Long COVID in Adults: United States, 2022.

[19] Ercoli T., Masala C., Pinna I., Orofino G., Solla P., Rocchi L., Defazio G. (2021). Qualitative Smell/Taste Disorders as Sequelae of Acute COVID-19. Neurol. Sci..

[20] Davis H.E., McCorkell L., Vogel J.M., Topol E.J. (2023). Long COVID: Major Findings, Mechanisms and Recommendations. Nat. Rev. Microbiol..

[21] Sharetts R., Moein S.T., Khan R., Doty R.L. (2024). Long-Term Taste and Smell Outcomes After COVID-19. JAMA Netw. Open.

[22] Taquet M., Geddes J.R., Husain M., Luciano S., Harrison P.J. (2021). 6-Month Neurological and Psychiatric Outcomes in 236 379 Survivors of COVID-19: A Retrospective Cohort Study Using Electronic Health Records. Lancet Psychiatry.

[23] Spudich S., Nath A. (2022). Nervous System Consequences of COVID-19. Science.

[24] Wong A.C., Devason A.S., Umana I.C., Cox T.O., Dohnalová L., Litichevskiy L., Perla J., Lundgren P., Etwebi Z., Izzo L.T. (2023). Serotonin Reduction in Post-Acute Sequelae of Viral Infection. Cell.

[25] Jegatheeswaran L., Gokani S.A., Luke L., Klyvyte G., Espehana A., Garden E.M., Tarantino A., Al Omari B., Philpott C.M. (2023). Assessment of COVID-19-Related Olfactory Dysfunction and Its Association with Psychological, Neuropsychiatric, and Cognitive Symptoms. Front. Neurosci..

[26] Taquet M., Sillett R., Zhu L., Mendel J., Camplisson I., Dercon Q., Harrison P.J. (2022). Neurological and Psychiatric Risk Trajectories after SARS-CoV-2 Infection: An Analysis of 2-Year Retrospective Cohort Studies Including 1 284 437 Patients. Lancet Psychiatry.

[27] Penninx B.W.J.H., Benros M.E., Klein R.S., Vinkers C.H. (2022). How COVID-19 Shaped Mental Health: From Infection to Pandemic Effects. Nat. Med..

[28] Shetty P.A., Ayari L., Madry J., Betts C., Robinson D.M., Kirmani B.F. (2023). The Relationship Between COVID-19 and the Development of Depression: Implications on Mental Health. Neurosci. Insights.

[29] Liu X., Zhu M., Zhang R., Zhang J., Zhang C., Liu P., Feng Z., Chen Z. (2021). Public Mental Health Problems during COVID-19 Pandemic: A Large-Scale Meta-Analysis of the Evidence. Transl. Psychiatry.

[30] Javed N., Ijaz Z., Khair A.H., Dar A.A., Lopez E.D., Abbas R., Sheikh A.B. (2022). COVID-19 Loss of Taste and Smell: Potential Psychological Repercussions. Pan Afr. Med. J..

[31] Speth M.M., Singer-Cornelius T., Oberle M., Gengler I., Brockmeier S.J., Sedaghat A.R. (2020). Mood, Anxiety and Olfactory Dysfunction in COVID-19: Evidence of Central Nervous System Involvement?. Laryngoscope.

[32] Paranhos A.C.M., Dias A.R.N., Bastos T.D.R., Rodrigues A.N., Santana K.H.Y., Dias L.H.A., Dos Santos L.P.M., Cerasi A.J., Mendes M.C.N., de Oliveira C.L. (2023). Persistent Olfactory Dysfunction Associated with Poor Sleep Quality and Anxiety in Patients with Long COVID. Front. Neurosci..

[33] Azcue N., Del Pino R., Saenz de Argandoña O., Ortiz de Echevarría A., Acera M., Fernández-Valle T., Ayo-Mentxakatorre N., Lafuente J.V., Ruiz-Lopez M., López de Munain A. (2024). Understanding the Olfactory Role in Post-COVID Cognitive and Neuropsychiatric Manifestations. Front. Psychol..

[34] Bochicchio V., Mezzalira S., Maldonato N.M., Cantone E., Scandurra C. (2023). Olfactory-Related Quality of Life Impacts Psychological Distress in People with COVID-19: The Affective Implications of Olfactory Dysfunctions. J. Affect. Disord..

[35] Dumas L.-E., Vandersteen C., Metelkina-Fernandez V., Gros A., Auby P., Askenazy-Gittard F. (2024). Impact of Post-COVID-19 Olfactory Disorders on Quality of Life, Hedonic Experiences and Psychiatric Dimensions in General Population. BMC Psychiatry.

[36] Rumeau C., Nguyen D.T., Jankowski R. (2016). How to Assess Olfactory Performance with the Sniffin’ Sticks Test^®^. Eur. Ann. Otorhinolaryngol. Head. Neck Dis..

[37] Beck A.T., Epstein N., Brown G., Steer R.A. (1988). An Inventory for Measuring Clinical Anxiety: Psychometric Properties. J. Consult. Clin. Psychol..

[38] Beck A.T., Steer R.A. (1993). Beck Anxiety Inventory Manual.

[39] Kroenke K., Spitzer R.L., Williams J.B. (2001). The PHQ-9: Validity of a Brief Depression Severity Measure. J. Gen. Intern. Med..

[40] Hummel T., Sekinger B., Wolf S.R., Pauli E., Kobal G. (1997). ‘Sniffin’ Sticks’: Olfactory Performance Assessed by the Combined Testing of Odour Identification, Odor Discrimination and Olfactory Threshold. Chem. Senses.

[41] Masala C., Saba L., Cecchini M.P., Solla P., Loy F. (2018). Olfactory Function and Age: A Sniffin’ Sticks Extended Test Study Performed in Sardinia. Chemosens. Percept..

[42] Masala C., Porcu M., Orofino G., Defazio G., Pinna I., Solla P., Ercoli T., Suri J.S., Spinato G., Saba L. (2024). Neuroimaging Evaluations of Olfactory, Gustatory, and Neurological Deficits in Patients with Long-Term Sequelae of COVID-19. Brain Imaging Behav..

[43] Oleszkiewicz A., Schriever V.A., Croy I., Hähner A., Hummel T. (2019). Updated Sniffin’ Sticks Normative Data Based on an Extended Sample of 9139 Subjects. Eur. Arch. Otorhinolaryngol..

[44] Mesholam R.I., Moberg P.J., Mahr R.N., Doty R.L. (1998). Olfaction in Neurodegenerative Disease. Arch. Neurol..

[45] Jung H.J., Shin I., Lee J. (2019). Olfactory Function in Mild Cognitive Impairment and Alzheimer’s Disease: A Meta-analysis. Laryngoscope.

[46] Kim B.-Y., Bae J.H. (2022). Olfactory Function and Depression: A Meta-Analysis. Ear Nose Throat J..

[47] Seighali N., Abdollahi A., Shafiee A., Amini M.J., Teymouri Athar M.M., Safari O., Faghfouri P., Eskandari A., Rostaii O., Salehi A.H. (2024). The Global Prevalence of Depression, Anxiety, and Sleep Disorder among Patients Coping with Post COVID-19 Syndrome (Long COVID): A Systematic Review and Meta-Analysis. BMC Psychiatry.

[48] Vilarello B.J., Jacobson P.T., Tervo J.P., Waring N.A., Gudis D.A., Goldberg T.E., Devanand D.P., Overdevest J.B. (2023). Olfaction and Neurocognition after COVID-19: A Scoping Review. Front. Neurosci..

[49] Bai F., Tomasoni D., Falcinella C., Barbanotti D., Castoldi R., Mulè G., Augello M., Mondatore D., Allegrini M., Cona A. (2022). Female Gender Is Associated with Long COVID Syndrome: A Prospective Cohort Study. Clin. Microbiol. Infect..

[50] Nouraeinejad A. (2022). Brain Fog as a Long-Term Sequela of COVID-19. SN Compr. Clin. Med..

[51] Krishnan K., Lin Y., Prewitt K.-R.M., Potter D.A. (2022). Multidisciplinary Approach to Brain Fog and Related Persisting Symptoms Post COVID-19. J. Health Serv. Psychol..

[52] van der Feltz-Cornelis C., Turk F., Sweetman J., Khunti K., Gabbay M., Shepherd J., Montgomery H., Strain W.D., Lip G.Y.H., Wootton D. (2024). Prevalence of Mental Health Conditions and Brain Fog in People with Long COVID: A Systematic Review and Meta-Analysis. Gen. Hosp. Psychiatry.

[53] World Health Organization (WHO) (2021). International Classification of Diseases, Eleventh Revision (ICD-11).

[54] Wei H.T., Kulzhabayeva D., Erceg L., Robin J., Hu Y.Z., Chignell M., Meltzer J.A. (2024). Cognitive Components of Aging-Related Increase in Word-Finding Difficulty. Aging Neuropsychol. Cogn..

[55] Baldo J.V., Arévalo A., Patterson J.P., Dronkers N.F. (2013). Grey and White Matter Correlates of Picture Naming: Evidence from a Voxel-Based Lesion Analysis of the Boston Naming Test. Cortex.

[56] Fonseca A.T.-D., Guedj E., Alario F.-X., Laguitton V., Mundler O., Chauvel P., Liegeois-Chauvel C. (2009). Brain Regions Underlying Word Finding Difficulties in Temporal Lobe Epilepsy. Brain.

[57] Harvey P.D. (2019). Domains of Cognition and Their Assessment. Dialogues Clin. Neurosci..

[58] Friedman N.P., Robbins T.W. (2022). The Role of Prefrontal Cortex in Cognitive Control and Executive Function. Neuropsychopharmacology.

[59] Twenge J.M., Joiner T.E. (2020). U.S. Census Bureau-Assessed Prevalence of Anxiety and Depressive Symptoms in 2019 and during the 2020 COVID-19 Pandemic. Depress. Anxiety.

[60] Jacobson P., Vilarello B., Snyder C., Choo T., Caruana F., Gallagher L., Tervo J., Gary J., Saak T., Gudis D. (2024). COVID-19 Olfactory Dysfunction: Associations between Coping, Quality of Life, and Mental Health. Rhinology.

[61] Al-Jabr H., Hawke L.D., Thompson D.R., Clifton A., Shenton M., Castle D.J., Ski C.F. (2023). Interventions to Support Mental Health in People with Long COVID: A Scoping Review. BMC Public Health.

[62] Pabel L.D., Murr J., Weidner K., Hummel T., Croy I. (2020). Null Effect of Olfactory Training with Patients Suffering from Depressive Disorders-An Exploratory Randomized Controlled Clinical Trial. Front. Psychiatry.

[63] Molnár A., Maihoub S., Mavrogeni P., Krasznai M., Tamás L., Kraxner H. (2023). The Correlation between the Results of the Sniffin’ Sticks Test, Demographic Data, and Questionnaire of Olfactory Disorders in a Hungarian Population after a SARS-CoV-2 Infection. J. Clin. Med..

