# Supplementary material for: Depression, Anxiety, and Neuropsychiatric Symptom Burden in a Longitudinal Cohort with Persistent Psychophysical Post-COVID Olfactory Dysfunction
Source: Brain Sci. 2024 Dec 19;14(12):1277. doi: 10.3390/brainsci14121277 (PMC11674626; doi:10.3390/brainsci14121277)
Supplement: Supplementary file 1 [file brainsci-14-01277-s001.zip › Table S1_brainsci.pdf]

Table S1. Characteristics and baseline scores of those who completed assessments at both time points (longitudinal cohort) versus those who were lost to follow up.

Measure at Baseline Assessment	Lost to Follow Up, N = 49[1]	Longitudinal Cohort, N = 48[1]	p-value
**Age**	42 (32, 64)	39 (32, 55)	0.4[2]
**Sex**			0.058[3]
Male	9 (18%)	17 (35%)	
Female	40 (82%)	31 (65%)	
**Education Status**			0.7[2]
Advanced Degree	22 (45%)	23 (48%)	
College	18 (37%)	22 (46%)	
High School	6 (12%)	3 (6%)	
No response	3	0	
**PHQ-9**	2.5 (0, 10.75)	4 (1.5, 8.5)	0.5[2]
**BAI**	5 (2, 12.25)	5 (1, 8.5)	0.3[2]
**TDI**	22.5 (19, 27)	26 (20.63, 28.63)	0.034[2]

[1]Median (IQR); n (%).
[2]Wilcoxon Rank Sum Test
[3]Pearson's Chi-Squared Test
