# Supplementary material for: Depression, Anxiety, and Neuropsychiatric Symptom Burden in a Longitudinal Cohort with Persistent Psychophysical Post-COVID Olfactory Dysfunction
Source: Brain Sci. 2024 Dec 19;14(12):1277. doi: 10.3390/brainsci14121277 (PMC11674626; doi:10.3390/brainsci14121277)
Supplement: Supplementary file 1 [file brainsci-14-01277-s001.zip › Table S2_brainsci.pdf]

**Table S2.** Prevalence of neuropsychiatric symptoms, depression, anxiety, and median BAI, PHQ-9, and TDI scores at baseline among all study participants (including those lost to follow up).

| Measure | Prevalence at Baseline, N=97[1*] |
|---|---|
| **Since COVID, do you now have more difficulty…** | |
| **Remembering conversations a few days later?** | |
| Yes | 25 (30%) |
| No | 57 (70%) |
| **Remembering placement of familiar objects?** | |
| Yes | 24 (29%) |
| No | 57 (71%) |
| **Finding the right words when speaking?** | |
| Yes | 40 (47%) |
| No | 44 (53%) |
| **Thinking clearly?** | |
| Yes | 28 (36%) |
| No | 48 (64%) |
| **Staying Focused?** | |
| Yes | 38 (48%) |
| No | 41 (52%) |
| **Since COVID, have you experienced…** | |
| **New or more frequent/severe headaches?** | |
| Yes | 25 (28%) |
| No | 63 (72%) |
| **Weakness in one or more parts of your body?** | |
| Yes | 23 (25%) |
| No | 67 (75%) |
| **Numbness or tingling?** | |
| Yes | 18 (20%) |
| No | 72 (80%) |
| **Changes in vision?** | |
| Yes | 19 (21%) |
| No | 71 (79%) |
| **Sense of dizziness, imbalance, or vertigo?** | |
| Yes | 26 (29%) |
| No | 63 (71%) |
| **Seizures?** | |
| Yes | 1 (1%) |
| No | 89 (99%) |
| | |
| **Beck Anxiety Inventory (BAI)** | |
| No Anxiety | 55 (67%) |
| Mild Anxiety | 14 (17%) |
| Moderate Anxiety | 9 (11%) |
| Severe Anxiety | 4 (5%) |
| No response | 15 |

| | |
|---|---|
| **PHQ-9** | |
| No Depression | 54 (59%) |
| Mild Depression | 15 (15%) |
| Moderate Depression | 8 (8%) |
| Moderately Severe Depression | 9 (9%) |
| Severe Depression | 5 (5%) |
| No response | 6 |
| **Overall Median (IQR) Scores** | |
| BAI | 5 (2, 1) |
| PHQ-9 | 4 (1, 9) |
| TDI | 24.75 (19.25, 27.75) |
| Threshold | 5 (2, 7.5) |
| Discrimination | 10 (8,11) |
| Identification | 9 (7, 10) |

[1] n (%); median (IQR).

*total participants reported for each symptom will not equate to 97 due to some participants choosing: (1) to not reply; or (2) "unsure".
