# Supplementary material for: Depression, Anxiety, and Neuropsychiatric Symptom Burden in a Longitudinal Cohort with Persistent Psychophysical Post-COVID Olfactory Dysfunction
Source: Brain Sci. 2024 Dec 19;14(12):1277. doi: 10.3390/brainsci14121277 (PMC11674626; doi:10.3390/brainsci14121277)
Supplement: Supplementary file 1 [file brainsci-14-01277-s001.zip › Table S3_brainsci.pdf]

Table S3. TDI median scores and evolution among longitudinal cohort (N=48).

Measure	Baseline[1]	Follow-Up[1]	p-value[2]
**TDI**	26 (20.63, 28.63)	28.75 (24, 33)	<0.001
Threshold	5.75 (2.25, 7.56)	7.75 (5.25, 10)	0.002
Discrimination	10 (8, 11.25)	11 (10, 12)	0.028
Identification	9 (8, 11)	10 (8, 12)	0.208

[1] Median (IQR).

[2] Wilcoxon signed rank test
