# Supplementary material for: Depression, Anxiety, and Neuropsychiatric Symptom Burden in a Longitudinal Cohort with Persistent Psychophysical Post-COVID Olfactory Dysfunction
Source: Brain Sci. 2024 Dec 19;14(12):1277. doi: 10.3390/brainsci14121277 (PMC11674626; doi:10.3390/brainsci14121277)
Supplement: Supplementary file 1 [file brainsci-14-01277-s001.zip › Table S4_brainsci.pdf]

Table S4. Correlations of TDI score versus BAI and PHQ-9 scores at baseline and follow-up among longitudinal cohort.

	**Baseline**[1]	**p-value**	**Follow-Up**[1]	**p-value**
**TDI and BAI**	-0.041 [-0.353, 0.259]	0.8	-0.185 [-0.488, 0.135]	0.3
**TDI and PHQ-9**	0.057 [-0.300, 0.390]	0.7	-0.222 [-0.529, 0.133]	0.2

[1] Spearman's rho [bootstrap 95% CI]
