# Supplementary material for: Depression, Anxiety, and Neuropsychiatric Symptom Burden in a Longitudinal Cohort with Persistent Psychophysical Post-COVID Olfactory Dysfunction
Source: Brain Sci. 2024 Dec 19;14(12):1277. doi: 10.3390/brainsci14121277 (PMC11674626; doi:10.3390/brainsci14121277)
Supplement: Supplementary file 1 [file brainsci-14-01277-s001.zip › Table S5_brainsci.pdf]

Table S5. Prevalence of neuropsychiatric symptoms, anxiety, and depression by gender at each time point.

	Baseline, N=97			Follow-Up, N=48		
**Measure**	**Men, N=26[1]**	**Women, N=71[1]**	**p-value**	**Men, N=16[1]**	**Women, N=32[1]**	**p-value**
**Since COVID, do you now have more difficulty…**						
**Remembering conversations a few days later?**						
Yes	3 (14%)	22 (35%)	0.067[2]	5 (38%)	9 (32%)	0.7[3]
No	18 (86%)	40 (65%)		8 (62%)	19 (68%)	
**Remembering placement of familiar objects?**						
Yes	2 (10%)	22 (36%)	0.021[2]	4 (27%)	10 (37%)	0.7[3]
No	19 (90%)	39 (71%)		11 (73%)	17 (63%)	
**Finding the right words when speaking?**						
Yes	8 (40%)	22 (39%)	0.5[2]	8 (50%)	20 (80%)	0.048[3]
No	12 (60%)	35 (61%)		8 (50%)	5 (20%)	
**Thinking clearly?**						
Yes	6 (30%)	32 (39%)	0.5[2]	5 (32%)	11 (42%)	0.5[3]
No	14 (70%)	23 (61%)		11 (68%)	15 (58%)	
**Staying Focused?**						
Yes	7 (37%)	31 (51%)	0.3[2]	9 (56%)	12 (43%)	0.4[2]
No	12 (63%)	30 (49%)		7 (44%)	16 (57%)	
**Since COVID, have you experienced…**						
**New or more frequent/severe headaches?**						
Yes	2 (10%)	23 (34%)	0.022[2]	3 (20%)	11 (38%)	0.3[3]
No	18 (90%)	44 (66%)		12 (80%)	18 (62%)	
**Weakness in one or more parts of your body?**						
Yes	5 (22%)	18 (26%)	0.7[2]	2 (14%)	7 (24%)	0.7[3]
No	18 (78%)	50 (74%)		12 (86%)	22 (76%)	
**Numbness or tingling?**						
Yes	3 (13%)	15 (22%)	0.3[2]	3 (20%)	9 (31%)	0.5[3]
No	20 (87%)	53 (78%)		12 (80%)	20 (69%)	
**Changes in vision?**						
Yes	2 (10%)	17 (25%)	0.096[2]	2 (14%)	5 (18%)	>0.9[3]
No	21 (90%)	51 (75%)		12 (86%)	23 (82%)	
**Sense of dizziness, imbalance, or vertigo?**						
Yes	3 (13%)	23 (35%)	0.039[2]	4 (25%)	8 (30%)	>0.9[3]
No	21 (81%)	43 (65%)		12 (75%)	19 (70%)	
**Seizures?**						
Yes	0 (0%)	1 (2%)	0.5[2]	0 (0%)	1 (4%)	>0.9[3]
No	25 (100%)	66 (98%)		15 (100%)	27 (96%)	
**Overall Median (IQR) Scores**						
BAI	5 (1, 8)	4 (1, 9.75)	>0.9[4]	5 (3, 2)	5 (1, 8.5)	0.7
PHQ-9	3 (1, 8)	4 (1, 9.75)	0.6[4]	4.5 (2.75, 8.75)	3 (1, 6)	0.2

[1] n (%); median (IQR).

[2] Pearson's chi-squared test.

[3] Fisher's Exact test.

[4] Wilcoxon rank sum test.
[*]total participants reported for each symptom will not equate to "N" due to some participants choosing: (1) to not reply; or (2) "unsure".
